# Working from Home During Covid-19: Doing and Managing Technology-enabled Social Interaction With Colleagues at a Distance

**DOI:** 10.1007/s10796-021-10182-0

**Published:** 2021-08-27

**Authors:** Banita Lal, Yogesh K. Dwivedi, Markus Haag

**Affiliations:** 1 University of Bradford, Richmond Road, BD7 1DP Bradford, UK; 2 Swansea University, Bay Campus, SA1 8EN Swansea, UK; 3 University of Bedfordshire, University Square, Luton, LU1 3JU UK

**Keywords:** Remote work, Technology-enabled social interaction, Working from Home, Social interaction, Covid-19

## Abstract

With the overnight growth in Working from Home (WFH) owing to the pandemic, organisations and their employees have had to adapt work-related processes and practices quickly with a huge reliance upon technology. Everyday activities such as social interactions with colleagues must therefore be reconsidered. Existing literature emphasises that social interactions, typically conducted in the traditional workplace, are a fundamental feature of social life and shape employees’ experience of work. This experience is completely removed for many employees due to the pandemic and, presently, there is a lack of knowledge on how individuals maintain social interactions with colleagues via technology when working from home. Given that a lack of social interaction can lead to social isolation and other negative repercussions, this study aims to contribute to the existing body of literature on remote working by highlighting employees’ experiences and practices around social interaction with colleagues. This study takes an interpretivist and qualitative approach utilising the diary-keeping technique to collect data from twenty-nine individuals who had started to work from home on a full-time basis as a result of the pandemic. The study explores how participants conduct social interactions using different technology platforms and how such interactions are embedded in their working lives. The findings highlight the difficulty in maintaining social interactions via technology such as the absence of cues and emotional intelligence, as well as highlighting numerous other factors such as job uncertainty, increased workloads and heavy usage of technology that affect their work lives. The study also highlights that despite the negative experiences relating to working from home, some participants are apprehensive about returning to work in the traditional office place where social interactions may actually be perceived as a distraction. The main contribution of our study is to highlight that a variety of perceptions and feelings of how work has changed via an increased use of digital media while working from home exists and that organisations need to be aware of these differences so that they can be managed in a contextualised manner, thus increasing both the efficiency and effectiveness of working from home.

## Introduction

Homeworking, increasingly known as ‘Working from Home’ (hereon referred to as WFH), amongst traditionally office-based workers is nothing new. WFH is where traditionally office-based workers now work from home by means of Information and Communications Technologies (ICTs). It has increased since the 1990 s due to developments in technology and the gradual uptake of flexible working styles by organisations (Dwivedi et al., 2020). The Covid-19 pandemic has presented numerous challenges for organisations across the world (Chakraborty & Kar, 2021) and has resulted in a huge shift from office-based working to WFH. As a result, studies that have questioned the uptake and usefulness of WFH over a period of three decades are called into question (e.g. Brocklehurst, 1989; Martin & MacDonnell, 2012; Pathak et al., 2015) as WFH becomes, for many, the new normal.

The number of employees who have started WFH has significantly increased and, in view of various positive experiences and investments that companies have made to enable WFH, the new WFH structures are expected to remain in place to some extent even beyond the pandemic (Schattenberg & Schneider, 2021). As of April 2020, very soon after the first lockdown began in the UK, 35.9 % of the employed UK population did some work at home: an increase of 9.4 % compared with 2019 (Office for National Statistics, 2021). As of August 2020, following the end of the first wave of Covid-19 in the UK, the figure increased to almost half of the UK population WFH five days a week or more. Also, in August 2020, Italy and Spain had approximately 60 % of workers WFH one day a week or more whereas in Germany, approximately 1 in 2 respondents said that in March 2020 they worked full-time or part-time from home (Statista, 2021). Although it is difficult to locate more recent figures to mirror the changing course of Covid-19, what is evident is that a substantial number of individuals were/are WFH as a result of the pandemic which has resulted in a shift in how employees conduct their work lives outside of the traditional workplace.

The lockdowns and the move away from working in a designated workspace have defined new categories of workers and revealed the ‘privilege’ of WFH, with some people working secured in their homes, some furloughed and others working as key workers (Fletcher and Griffiths, 2020). Whilst having a job to do and being able to work from home is indeed a privilege, there is a lack of empirical evidence explaining how homeworkers are adjusting to – and subsequently getting on with - work in their new, full-time home-workplace. There are some insights provided by previous literature: for example, there is extant literature on WFH in fields such as Human Resource Management, focusing on issues such as remote working and increased employee flexibility, wellbeing and productivity (Grant et al., 2013), stress, job satisfaction and costs (Nakrošienė et al., 2019), as well as homeworkers’ ability to segment and balance their work-home lives (e.g. Kreiner et al., 2009, Tietze et al., 2009, Park et al., 2011). However, it can be argued that the pandemic is a unique situation which may result in unique implications: for many, working from home is sudden and has resulted in the upheaval of existing daily work practices.

Even with reference to previous literature, there are still issues that remain under-explored within the context of WFH in general. Working is not solely the completion of a task in isolation but requires - at least in a wide range of circumstances - a high level of social interaction. How homeworkers are able to manage their social interactions with colleagues via technology when working remotely – so to avoid feelings of social isolation - is under-researched. Given that so many people have started to work from home and the increasing media attention on the impact of social isolation, research on this topic is both timely and necessary.

It is a basic human need to want to associate and identify with others via long-term, positive relationships: not having face-to-face social interaction can affect communication and camaraderie, interpersonal networking and the sharing of work-related information and gossip which has the effect of enabling employees to create identification with the company (Lal & Dwivedi, 2009). The World Health Organization asserts that social well-being is an integral part of health: “Health is a state of complete physical, mental and social well-being and not merely the absence of disease or infirmity…The enjoyment of the highest attainable standard of health is one of the fundamental rights of every human being” (World Health Organization, 2021). With this definition in mind, within the traditional workplace, there are several social exchanges that may take place between an individual and (a) the organisation, (b) their supervisor, and (c) their work group (Cole et al., 2002): such interactions are a fundamental feature of social life and the workplace relationships that employees engage in with colleagues at all levels are important in shaping their experience of work (Collins et al., 2016). The situation regarding social interactions with colleagues clearly changes with WFH where the proliferation of more advanced ICTs since the early days of WFH suggests that homeworkers should be able to retain communication via different technological means, allowing individuals to feel more ‘socially present’ (Dwivedi et al., 2020). However, whether this is done and how it is done is not well understood. Presently, popular press articles, short research papers and opinion pieces have been written which provide little scientific import or practical value (Davison, 2020). Thus, the aim of this paper is to investigate *how homeworkers engage in technology-enabled social interactions with colleagues when working from home.* In line with this aim, there are two research objectives: (i) to explore the methods used to retain social interaction, and (ii) to identify any challenges/hindrances to social interaction when working from home.

This understanding is important given that the pandemic has changed – and is expected to continue to change – how work is conducted in the future which ultimately impacts how employees conduct relationships and interactions with colleagues. A prudent understanding and management of the latter is likely to improve both the experience and potentially the efficiency of working together as well as reducing the potential of employees feeling socially isolated. The study was not restricted to a specific country or context: given the exploratory nature of the research, the sample covers several countries and work contexts in order to get the widest possible insight into the study problem and to identify whether there are any similar patterns of behaviour amongst participants, e.g. amongst participants from particular geographical regions.

The paper is structured as follows: an overview of the literature in relation to WFH and social isolation is firstly presented followed by the methodology deployed in this study. This is followed by the key findings, a discussion which includes the theoretical contributions, practical implications of the study, and the limitations and recommendations for future research, followed by the conclusion.

## Literature Review

### Understanding ‘Social Interaction’

“Social interaction is the most elementary unit of sociological analysis” (Turner, 1988, p.14) and the study of behaviour - and how behaviour is implicated in the process of social interaction - is discussed at considerable length within Sociology and Psychology. Such disciplines explore how groups are formed and their interactions in detail. Social interaction has naturally been a focal point within Sociology and Psychology where the role of social interaction has been explored in understanding a range of topics. There is a specific focus on who interacts with whom and why in urban, industrial society where answers to “the “who” ordinarily appear categorically, friendship milieus and various degrees of kinship being distinguished, with frequency of interaction as the primary indicator of involvement” (Adams, 1967, p.64). Thus, individuals choose to remain in contact with others with whom they share social bonds and affection/liking (Adams, 1967). Whilst useful, theories in Sociology and Psychology delve into details on micro social processes and concepts such as language, rituals and institutional frames. There is relatively little understanding of how technologies feature in day-to-day organisational conduct and interaction in general (Heath et al., 2003), let alone in the WFH context.

Furthermore, homeworkers in the current context are not typically ‘virtual teams’ wherein members are geographically distributed and have often not met each other in person (Tan et al., 2000). Virtual teams are primarily connected via ICTs and “function independently of organizational boundaries, geographical locations, and time zones while striving effectively to reach the team-specific goals” (Lilian, 2014, p.1253). Virtual teams exist in organisations that have decided to have a different structure/form and working practices – there has been a reasoned decision made whereas with WFH during the pandemic, there is no other choice but to work remotely. It is, nevertheless, useful to note that studies on virtual teams have found that despite the availability/reliance on ICTs, the teams can become ineffective because electronic communication does not facilitate the building of shared understanding amongst the virtual team members (Tan et al., 2000; Orhan et al., 2016) and that in virtual settings where the dependency upon technology is high and in-person face-to-face contact is low, employees are likely to experience decreased job satisfaction, commitment, identification and increased workplace social and physical isolation. Homeworkers in the current context are a bit of an oddity: not only are they entering new working practices, but also have no established shared understanding and norms in terms of how communication is done and how social relationships are maintained when working remotely.

### Remaining Connected Whilst Working from Home During Covid-19

The role of technology is increasing (Kar et al., 2019, 2021; Elbanna et al., 2020) and has come to the forefront during the pandemic as online applications have enabled the continuity of personal and business activities (Papagiannidis et al., 2020). Presently, in the Information Systems/Management area, there is much speculation, expert opinion and suggestions for research and theory in relation to the longer-term implications of Covid-19. The initial stage of immediate panic and high levels of change required to adapt working practices has now passed: organisations and employees have managed to adapt working practices to function remotely using collaborative tools such as Microsoft Teams and Zoom (Barnes, 2020). Inevitably, change has occurred as employees are now separated from their office environment and colleagues. However, how this change has impacted upon employees’ ability to maintain social relations with colleagues and subsequently manage feelings of social isolation remains empirically unsupported. This is despite there being recognition that increasing digital social interactions risk “dehumanizing human-human interactions or unduly humanizing technology-human interactions” (Seetharaman et al., 2020, p.530).

The lockdown has resulted in more people using the internet and internet-based services for communication, interaction and work purposes from home: cities such as Bangalore in India have witnessed a 100 % increase in internet traffic whereas the usage of video conferencing applications such as Zoom has increased ten-fold (Dé et al., 2020) resulting in changed work practices (Barnes, 2020). Since video conferencing platforms are widely available, it is suggested that most users would not have faced major issues using these (Papagiannidis et al., 2020). Furthermore, Kodama (2020) asserts that video communications/conferencing is a high-potential method of communication that is very useful for conveying image information and personal expressions. However, the author states that there is limited research on IT application-related functions such as video conferencing tools and their impact on business and social domains which requires further research. This is particularly relevant because collaboration systems affect an organisation’s processes, knowledge-sharing amongst employees, knowledge creation and innovation (Kodama, 2020).

It is suggested that employees across the world have experimented using digital work tools such as video conferencing for ‘virtual morning teas’ and ‘after work (social) zooming’ (Richter, 2020). However, how such an application can help to alleviate feelings of isolation remains unknown. In addition, there is the potential challenge of maintaining the organisational culture when employees work at a distance from one another (Barnes, 2020). Thus, how well technology can be used to maintain communication for work and social purposes, as well as maintain an organisational culture, is unclear. Thus, as is becoming increasingly apparent, there is a lack of clarity regarding numerous issues.

The use of video calling is called into question as authors such as Davison (2020) suggest turning off the video during a video call can help individuals in meetings as it reduces the number of cues they need to process as well as noticing video-audio lags, even though some of the paralinguistic cues would disappear. Further, Davison states how in-person interaction – in his case, a research visit – is more than just a meeting since it involves richer interactions that take place aside from the meeting, such as “one-to-one conversations, brainstorms, insights, and the exchange of ideas, lubricated with laughter, intellectual spice and good cheer” (Davison, 2020, p. 2). In the absence of face-to-face meetings, it is unclear how technology can enable this level of interaction, both work-related and jovial in nature, to continue. Echoing the questions raised by Kodama (2020), whether technology can effectively replace in-person interactions is questioned.

Fahey and Hino (2020) assert that digital communication and social media platforms have helped to support citizens’ mental health and their social relationships during social distancing and isolation. Further, Nabity-Grover et al. (2020) explain how people have responded to, and managed the negative implications of, social distancing by spending more time on social media platforms such as Facebook, Instagram, Weibo and TikTok in order to remain connected to friends, family and colleagues. However, lack of empirical evidence makes it difficult to develop an understanding of how this is happening, particularly in relation to remaining connected to colleagues via social media. Considering there are several reports in the popular press that some firms will allow some employees to continue working from home ‘forever’, it is suggested that as employees have adapted and adjusted to WFH and online meetings and transactions, WFH will become a norm within organisations as opposed to an exception. Again, there are nuances that need to be addressed and it is becoming increasingly clear that there is no one-size-fits-all approach (Seal, 2020). Thus, research on WFH is important for the post-pandemic period (Dé et al., 2020).

### Existing Research on WFH and Social Isolation

In terms of what we can learn from existing literature on WFH, a considerable amount of research has examined how homeworkers have tackled the issue of no longer having spatial and temporal distinctions between their work and home lives by reconstructing these boundaries in the home-work place (Desrochers & Sargent, 2003). There has also been some research on how homeworkers construct boundaries in relation to mobile devices (Cousins & Robey, 2015; Hislop et al., 2013, 2015). However, lesser-researched topics focus on how homeworkers are able to manage their social interactions with colleagues in order to avoid feelings of social isolation in the absence of the day-today socialisation and relationship-building communication shared in the traditional office space (Lal & Dwivedi, 2009).

The topic of WFH and social interaction/isolation is, at best, briefly included as part of wider studies examining the implications of flexible working. It is acknowledged that relatively little research has focused on social support relationships between flexible workers and their colleagues, whether they are also flexible workers or based in a traditional office setting (Collins et al., 2016). For example, in a study by Maruyama and Tietze (2012), it was found that half of respondents felt that no professional/social interaction was a pre-telework concern (‘telework’ is often used synonymously with ‘WFH’). However, the study did not expand on actual experiences of such interaction once the change in work style had been made. Studies that do provide some explicit insights into homeworkers and feelings of social isolation are very limited in number: examples are provided below.

In a study of sales staff working from home (Harris, 2003), it was found that 63 % of homeworkers said they felt isolated since starting WFH, stating that they felt forgotten and left to ‘muddle through’ when working remotely. Lack of in-person interaction resulted in a reduction in the speed of problem-solving and knowing what was going on as it became more difficult to share experiences. Further, infrequent team meetings were described as formal with full agendas and little scope for informal discussions with employees feeling invisible.

Mann and Holdsworth (2003) found that 67 % of individuals working from home acknowledged feeling loneliness, compared to 0 % of office-based counterparts. The lack of face-to-face communication was an issue: there was no-one to talk to at the end of a difficult day and homeworkers would go out to the shops to have face-to-face interaction with somebody. Further, increased use of technology reduced feelings of belonging with the company and homeworkers lacked social support which could give rise to other emotions such as feelings of insecurity and lack of confidence in their abilities. The authors assert that the social interaction of the workplace is “utterly important” and homeworkers feel the stress of separation from colleagues and social banter within the office (p.208).

Lal and Dwivedi (2009) found that homeworkers were provided with work mobile phones and social interaction via these devices could be managed to keep work and home lives distinct. Three main types of information were exchanged: general gossip about other colleagues, information about developments/changes in the company and advice on how certain work tasks could be completed. The study highlighted that homeworkers would seek social interaction with family members to compensate for the lack of social interaction with colleagues. Furthermore, when communicating with colleagues, the sender of the message thought carefully about the best mode of communication (e.g. a SMS was less intrusive at the weekend than a call), and that a small network of close colleagues – which can be described as ‘human colonies’ akin to animal colonies (Porra et al., 2020) - was key both for work support and social interaction. Very few studies investigating how homeworkers use/manage their technologies, particularly mobile technology, in the home-work place have been conducted since this study (Hislop et al., 2015).

Considering the above, what we can deduce from previous studies is that there is little doubt that maintaining social interactions with colleagues is important on many levels. Technology offers promise in terms of enabling homeworkers to retain some level of social interaction with colleagues: the current situation highlights how technological advancements provide potentially richer and varied forms of communication with colleagues. Thus, theoretically speaking, if such communication media are available and individuals are proficient in using them, feelings of social isolation can be reduced despite the limitations of non-in-person interaction. However, as aforementioned, empirical evidence in terms of how this is done, what technology is used and how social relations are maintained is limited. Thus, there remains scope to investigate the application of advanced collaborative technology to support teams and organisations from varied contexts (de Vreede et al., 2016) including that of WFH.

The following section provides details of the methodology employed in investigating how homeworkers engage in technology-enabled social interactions with colleagues when working from home.

## Methodology

This study adopted an exploratory, qualitative approach utilising the diary-keeping technique. Due to the exploratory nature of this study, a particular theoretical lens was not used with the view to developing a framework informed by the findings. An interpretive perspective was adopted which emphasises the human role as social actors in a setting (Saunders et al., 2009): this approach appreciates that reality becomes multiple, subjective and mentally constructed by individuals (Crossan, 2003). It considers social factors as well as the natural context/settings and thus helps the researcher to uncover the complex reality of human beings and their organisational processes (Klein & Myers, 1999). This aligns with the aim of this research which is to understand how individuals go about engaging in social interaction via means of technology when WFH.

### Data Collection

Participants were requested to complete daily diary entries over a period of ten working days. Not only did this offer an insight into participants’ everyday lives as it naturally unfolded (Neupert & Bellingtier, 2018), but respondents were able to record responses on the day. Diaries were kept between May and June 2020 during the height of the first lockdown. There were six standard questions that participants had to consider daily: (i) their working hours; (ii) how they felt personally/professionally while working remotely; (iii) whether they had any social interaction with colleagues; (iv) if ‘yes’ to (iii), then the method of communication used, information exchanged and time of interaction; (v) whether they did any non-work-related activities to keep positive, and (vi) any other comments they wanted to make. The questions were intentionally kept simple so participants did not perceive completing them a daily chore, to reduce the likelihood of participants dropping out of the study and, owing to the exploratory nature of the study, enabled participants to record what was important for them. Participants were also asked to complete an additional information sheet which provided details such as demographic data.

Although diary studies tend to be used more commonly in areas such as social and personality psychology (Nezlek, 2012) and are less commonly used in Business and Information Systems research, there were key reasons as to why diary studies were selected for this study: diary methods allow researchers to gather data in participants’ natural life contexts such as at home or in the workplace. This data can take the form of events, behaviours, feelings and thoughts (Ohly et al., 2010). Thus, diary methods were used to get an insight into participants’ day-to-day behaviours in terms of social interaction with colleagues including how the interaction takes place and the type of information exchanged. Diary studies enable data to be collected on a daily basis and in a way that is not possible using traditional designs (Bolger et al., 2002) such as surveys and interviews where data tends to be collected at one point in time. Recording responses on the day enabled participants to document any particular feelings/thoughts/behaviours they experienced and eliminated reliance upon memory as is the case in interviews and surveys. Having a record of daily experiences also helped the researchers to build a better picture of the participants as each gave new information every day which helped to develop more of a context to their responses. Some respondents who worked during the weekend documented their experiences on these days. The drawback of a diary study is that participants may forget to record some information in their daily entries (Sohn et al., 2008). However, this can be regarded as a limitation with other methods as well where participants may not be able to recall certain information until after data has been collected.

Participants were recruited using a snowball sampling technique. This entailed the researchers initially contacting individuals they knew who had recently transitioned to WFH due to the pandemic, and then these participants recommending other potential participants. Diaries were completed by a total of 29 participants. Participants were also asked to complete an additional information sheet which provided details such as demographics. Although there are no specific rules in terms of the number of participants required for qualitative research, it is acknowledged that the sample size used in qualitative research methods is generally smaller than that used in quantitative research methods since qualitative research focuses on developing an in-depth understanding of a particular phenomenon rather than making generalisations to a larger population (Miles & Huberman, 1994). Further, based on an examination of 83 qualitative studies in leading IS journals, Marshall et al. (2013) recommend that single case studies should generally contain between 15 and 30 interviews. Applying the same principle to this study where the rich data provided in the diary entries can be likened to the rich data provided in interviews (if not richer), this suggests that the 29 participants in this study meet this recommendation.

### Data Analysis

Data was analysed as follows, following guidelines by Miles and Huberman (1994): a contact summary sheet was created for each participant. This entailed going through the ten days’ diary entries in order to summarise the data provided. Summary notes were written up in relation to the six questions that participants completed in their day-to-day diary entries. The sheet enabled the researcher to reflect on the data, helped with the coordination as there was more than one researcher analysing the data, and helped the researchers to reorient themselves to the contact when returning to each participant’s data. Codes were applied to the data which was the first stage of analysis: there was no starting/initial list of pre-existing codes prior to the data collection that researchers were trying to match to participants’ data as this study was more exploratory in nature and there is currently limited empirical data which can be used to formulate pre-existing codes. On the summary sheet, researchers documented the summary data, respective codes and the salient points to provide explanations/definitions of the codes, alongside the page numbers on which that code appeared. This allowed for easier retrieval of specific data. A complete list of codes was also created together with details of which respondents these codes related to. Thus, assigned codes could be cross-checked with the complete list of codes and individual summary sheets of participants. Codes were revised as necessary.

Having a record of daily experiences and the additional information sheet helped the researchers to build a better picture of the participants which helped to develop more of a context to their responses. Causal diagrams were created which helped to consider each participant’s context and experiences. Once the researchers were able to understand more of the context in which the participant worked using the causal diagram and the additional information sheet, it was possible to interpret the codes and data in the given context. This led onto pattern codes being developed. Demographic data was organised using a matrix which enabled the researchers to gain a snapshot view of key data for all participants in one spreadsheet. This also enabled the researchers to develop a better understanding of the participants as well as explore potential patterns between certain demographics and causal diagrams.

### Validity and Reliability

Two researchers were involved in the entire analysis process outlined above which ensured shared understanding and agreement of the codes, causal diagrams, relationships between variables and the identification of key themes from the findings. This process ensured inter-coder reliability. For example, agreement on codes used helped to determine clarity; internal consistency, the meaning assigned to them and code-recode reliability resulting in both intra- and inter-coder agreement. By qualifying a pattern code – identifying the conditions under which it holds – this helped to verify the pattern and strengthen its external validity. Including participants from different countries helped to affirm the validity of the patterns in different settings. Check-coding about halfway during the data collection/coding was useful to ensure the codes retained the same meaning/could be reconsidered. The use of causal diagrams and matrix also helped to review the initial findings and to see how well-supported preliminary findings/patterns were, to note any inconsistencies and contradictions and for making comparisons which, in turn, helped assure the internal validity of the emerging patterns and subsequent interpretation of the results. The key findings are presented in the following section.

## Findings

There were 15 males and 14 females who participated in this study. 17 participants were located in the UK, four in India, three in the USA, two in Germany and one each in Nepal, Canada and Luxembourg. Occupations of participants were varied and included: Web Administrator and Digital Marketer, Software Developers, Academics, Science Policy Analyst and Sales Account Executive. 19 participants used either solely personal devices or personal plus work devices provided by their company for work. 10 participants used only devices provided by the company. Nine participants had never worked from home before the pandemic, whereas 20 participants had. Table 1 presents the number of participants as per their age groups.

**Table 1:** Age groups of participants

Age group (years)	Number of participants
25–34	16
35–44	6
45–54	3
55–64	3
65–74	1
Total	29

**Table 2:** Length of time at current organisation

Time working at current organisation (in years)	Number of participants
< 1	6
1–4	15
5–9	2
10–14	3
15–19	1
20–24	1
25+	1
Total	29

Inevitably, it is important to consider the context in which the participants worked. All participants bar two mentioned having a heavy workload. The uncertainty of current events was something that weighed on participants’ minds. There was no correlation between demographics such as gender, age group, where they lived and feelings of social isolation or levels of social interaction.

### The Meaning of ‘Social Interaction’

Participants were asked whether they had any social interaction with colleagues on each day they kept a diary. The researchers intentionally did not define what ‘social interaction’ was in order to understand how participants defined it and what type of information was exchanged. In most cases, social interaction was defined by participants themselves as *any type of communication with colleagues*, whether this was work or non-work related. Participants (referred to as ‘P.1’ for ‘Participant 1’, etc.) tended to communicate via Zoom, Microsoft Teams, Slack, and Google Chat/Meet. These were used for individual chat, group chat, video and voice calls related to work and non-work. Other communication methods were also used, although to a lesser degree, including: email, telephone, WhatsApp, Facebook, FaceTime, Xbox Live and SMS.

52 % of participants stated that social interaction was mainly for work purposes, such as online team meetings. In response to the diary question asking participants what social interaction they had that day, a typical response was:

“I had a call with my boss, a call with one of my juniors, and have spoken to all other members of my team alongside many of my other colleagues who work in our other department. The vast majority of this was work related (I would say 98 %!) - with a couple of work-related jokes involved.” (P.24).

49 % of participants stated that informal, brief social interaction was built into the beginning/end of meetings:

“[Meetings are about] Mainly work but also a few minutes of social chats on how we spend our days, tips on keeping fit as well as introducing kids to colleagues and their kids on Zoom” (P.2).

“Had a couple of Zoom meetings with colleagues. Prior to meeting starting had a few min[ute]s chat.” (P.23).

There were very few participants who had time specifically dedicated for social interaction, for example:

“I also had a coffee chat with another department I work with so that we have that level of social interaction as we have while at work…discussing what we are doing to stay sane and active. And catching up what we did the previous week. Also we see each other’s pets or show our shopping.” (P.12).

Types of information exchanged during social communication ranged from discussing the history of spaghetti carbonara (P.26), showing each other their children (e.g. P.28), pets and shopping (P.12), Covid-19 (P.3), changes in the company (e.g. P.19) and online gaming with colleagues such as playing Xbox (P.11) and poker (P.1).

### A Reduction in Social Interaction

There was no doubt that social interaction had reduced as a result of working from home:

“I feel like the proportion of casual/personal conversations I am having with colleagues is a lot smaller when working from home (i.e. nearly all conversations are about work matters). For example, today all messages I exchanged with colleagues were about work. Without the casual conversations to break the day up, it can give the workday a more serious feel.” (P.14).

Others stated that actual non-work social interaction had declined, even when efforts were made to maintain it whilst working from home:

“…we used to have zoom social session at the beginning on the lockdown but its fading now…” (P.18).

Only five respondents said that social interaction had either stayed at the same high level as when working in the office, or had actually increased since working from home full-time, which has been positive for their group bonding. Where social interaction had increased, one respondent stated that this was due to more company efforts to engage employees and some days were not just filled with work meetings, but also social interaction built into work emails and specific social activities. For example, one participant who had worked at their organisation for over fifteen years stated:

“I must say that I think I get more interaction with people now than when in the office… and enjoyed it today. I enjoyed the email chat with the colleague in Singapore. And the coffee morning was more fun today. We had quiz like what advert is this for by watching the few seconds and also movie clips and say the title. [I] Was rubbish at both but was fun.” (P.2).

Regarding the two participants for whom social interaction remained as high as in the office, they made notable efforts to maintain this. For example:

“It was a very busy day and we are always chatting on the side in Microsoft teams like we would at work. We have created groups with different people from work so every[one] feels involved and isn’t left out… as it was Friday we again had our coffee chat….We also had a Spring Fling arranged which is an event arranged every year in college. We all decided to dress up, have music and bring our drinks and snacks on the virtual meeting we had at the end of the day and gossip….” (P.12).

#### Absence of Face-to-face Interaction

The findings suggest that on the whole, at some point along their diary-keeping journey, the majority of participants stated that they missed face-to-face interactions with colleagues. There was only one participant who stated that he felt no impact professionally or personally working from home as he was happy to just focus on his work *(P.10)*. Working remotely clearly impacted upon the experience of work of which social interactions, often spontaneous and supportive in nature, are a part. Irrespective of whether the participants overall enjoyed or did not enjoy WFH, a number stated in their diary entries that they missed face-to-face interaction:

“It didn’t affect me professionally, but personally I missed a chat today.” (P.26).

“I do miss my office, interacting with colleagues, and just the separation between Office and home… This human interaction, work chats, corridor talks and laughs…are the best part of my job which I miss.” (P.18).

Some respondents documented their feelings regarding being apart from colleagues on a daily basis. For example, the following excerpts are from the diary entries of P.29:

Day 2: “I missed some of my work friends a bit today. The weather was nice so I thought about how it would be to meet them and that I can’t because I don’t live in London where they live.”

Day 5: “I missed them a lot today, mainly from a friendship perspective, but also from a social team perspective”.

Day 6: “Very much missed my team today, would have loved to see them. It made it hard to concentrate on work sometimes.”

Furthermore, in the absence of lunch and tea breaks in the office which would normally be with colleagues, 1 in 4 participants stated that their social interaction during breaks came from another person living with them. For example:

“Small conversations with partner who is also working from home at lunch time.” (P.23).

“I spoke in person to my partner who also works within the same [organisation]. This was a social discussion at lunch as we work in different rooms all day – as we would at [work].” (P.21).

#### Balance Between Social Interaction and Quiet Time

52 % of participants said they appreciated the ability to do focused work whilst working remotely that allowed them to be more productive:

“[Working at a distance from colleagues affected me] in a positive way, I can concentrate on my corrections.” (P.17).

“I worked quietly behind the scenes trying to catch up with paperwork today. Professionally I got lots done. Personally, I felt good to be able to hide and relax into have a slightly easier day.” (P.21).

A number of participants said that face-to-face interactions were actually a distraction that could affect their productivity:

“[I] did not miss working at a distance from my team as there are likely to be less distractions.” (P.5).

“I had quite a lot of work to do, so it was helpful to not have distractions that being in an office brings, and take breaks when convenient.” (P.14).

Again, the benefits of working from home could potentially outweigh the drawbacks:

“I do miss some face-to-face discussion. On the other hand, working without walk-in traffic does not feel too bad” (P.15).

The findings suggest that participants need to establish a balance between social interaction with colleagues and quiet, focused time for work/personal time:

“There was a lot of social interaction today to the extent that I didn’t feel like I had much time to myself to recharge which was exhausting. I started the day positively, but I’m glad to put it behind me.” (P.11).

### Deciding with Whom to Interact

As well as balance, it was important to decide with whom to interact. For example, it was straightforward to become part of an online social space with other colleagues. However, there were issues when there was a lack of mutual interest. P.20 was one of four people who stated that they used WhatsApp to remain socially connected to colleagues; participants generally did not connect with colleagues on other social media platforms:

“I find that some of the content that is shared [on the work WhatsApp group] is really not for me…I like that my colleague set up this group for social interaction but I think we are finding that people’s personalities are so different and there is a reason why we are just colleagues and not friends… I decided to leave [the group] and I am so happy. No more random content coming in where you feel compelled to comment… And far less disturbances during personal time.” (P.20).

There was also some apprehension in terms of joining online social events with consideration given to who else would be present:

“There are some zoom coffee sessions scheduled…no one talks about work in those sessions but I never joined them as they are voluntary. We anyways have so many online meeting, no personal interaction, I didn’t want to go for another one even if it was non work related. I also did not feel comfortable as senior management team was part of it, when we never had social interaction with them ever before lockdown then how can it be a social interaction, in a comfortable and informal environment during lockdown. I excused myself.” (P.18).

The importance of interacting with close colleagues was emphasised by over a third of participants: it was important for sharing news about the organisation, general gossip and other information, as well as providing a support network for when individuals felt overwhelmed with working from home and/or work pressures:

“An informal catch up with my close colleague via messages, helped me to realise not to let my workload, tasks or teams I work with get the better of me.” (P.5).

Two participants had new team members join their team/organisation and commented:

“I’ve been thinking about the experience the joiner to my team that I have been assigned to look after has had so far. In a normal office environment, I think he would have made social connections with more people in the team. Since we talk regularly over instant messenger or video call, he is less likely to contact others (for example, if he has a work-related question) in a socially distanced work environment. So I think these working conditions are narrowing his work social circle relative to what he would otherwise have.” (P.14).

“We have had several new members joining my team since the lockdown. This must feel challenging for them, but we’ve done what we can with introductions and creating more informal spaces on teams, like a ‘café’ where people can join in and have a chat on their coffee break.” (P.4).

From the perspective of a new member of staff, there were nine individuals who had worked in their present organisation for less than one year. Eight participants did not highlight any particular issues whereas one stated:

“I’m frustrated and annoyed…I’ve just been ‘dumped’ a lot of work, and there is really no one who I feel I can talk to at the moment. I’m tired and am not going to do more than I need to do today…. I can’t stop thinking about how much work I have to do. I can’t just leave the work and come back tomorrow. It’s tough.” (P.8).

### Work and Communication Overload

Technology was clearly important for both work-related and non-work-related communication. The issue of communication overload was something that the majority of participants stated in their diary entries. All participants were heavily reliant on technology for online meetings with colleagues. Some stated that they were too busy with work to have any social interaction with colleagues:

“Today, we could not interact much due to workload.” (P.28).

“There was a social activity within our unit today, an online scavenger hunt, but I wasn’t able to join as I needed to catch up on work and didn’t feel able to take the time out. This was a shame but I also don’t currently have the energy for huge online social activities with colleagues.” (P.30).

The amount of work-related communication that participants now had with colleagues was often high and often had various negative consequences. This was something highlighted by approximately half of the respondents. Communication overload resulted in less productivity during the working day, thus leading to working longer hours:

“Certainly, due to lockdown, the hustle and bustle of travelling to work (especially in London tubes/underground) has been removed from my routine which has given a few extra hours to focus on my work and be more productive. However, in my opinion that increase in productivity gets nullified or even sometimes reduces further [because since working from home, there has been a] significant increase in time dedicated for meetings, skype, video calls…This in turn leaves lesser time to focus on your own work for which one needs thorough concentration and hence end up sitting back late or outside business hours to get it completed.” (P.27).

Whilst 1 in 4 respondents stated that video calls were a good way of communicating as it enabled individuals to see one another, there were mixed responses about them. For example, P.4 highlights the usefulness of being able to see colleagues:

“Today I have been reflecting on the value of face-to-face and what that means. To me video calls and in person are of similar value in terms of my wellbeing and positivity after the interaction, followed quite a long way afterwards by phone calls… there is something to me there about the visual interaction and it’s importance.” (P.4).

However, when she had a heavy workload, the same respondent stated:

“I’ve noticed I’m becoming a bit more anxious on days I expect to have a video call.” (P.4).

1 in 5 participants stated that video calls were draining, owing to the number of hours spent on them, often daily. Communication overload meant that some participants had to recuperate following hours on video calls and long hours at the computer were having a toll on their health:

“I’m sat at the computer for so long that I’ve been feeling it over the last few weeks…it was a long day and I felt tired after back to back video calls throughout the day…[I have experienced] mood changes, in terms of increased stress and also the physical effects – such as my shoulders and neck tensing up” (P.30).

Multiple meetings meant that some participants were working virtually non-stop during working hours:

“Meetings back to back. 10 min lunch break…The biggest impact while WFH is I cannot even spare a few minutes to call my Bank to sort out what I want. Day packed with meeting from start to finish.” (P.19).

It was mandatory for the majority of participants to remain connected via video and audio during meetings. This could bring its own challenges and feelings of awkwardness. For example, in a working group meeting of fifteen colleagues *“the spaces usually filled with discussion in person were largely silent though, as has been the case for some of the larger group meetings. Screen sharing.” (P.4)*.

#### Managing Communication Via Multiple Platforms

The availability of different communication methods via different technology platforms also meant that participants had to manage these platforms which could prove challenging:

“Awareness of the use of these new social and collaboration platforms – Microsoft Teams, Skype for Business, WebEx, Zoom, Google Duo etc…can be quite overwhelming at times when people are trying to connect to you through different modes.” (P.27).

Different communication methods also meant that individuals were more contactable which increased anxiety:

“My day was busy and it felt a little chaotic as my manager phoned me several times throughout the morning with new tasks he wanted me to do urgently and I already had a lot to be getting on with so it was a bit stressful and difficult to manage.” (P.12).

“My team leader gave me a task and after 2 hours he kept sending me messages via teams on updates. At some point I felt like I was going crazy.” (P.7).

### Issues Relating to Social Interaction

Participants also highlighted issues relating to getting their message across via technology, stating that face-to-face interaction was better for avoiding miscommunication, to convey feelings to colleagues, for discussing sensitive topics such as deaths and job progression, and for ‘reading’ their state of being:

“I needed to ask [a colleague to do a task] and I haven’t seen him since he was ill. I worry that without that visual contact and being able to judge if he is in a place to cope with anything extra to do I might be the straw that broke the camel’s back. We judge how people are and use emotional intelligence when we see and chat with them on a regular basis. Judgements are made in the dark when working in this remote manner.” (P.21).

P.30 stated that although using Teams video call was a good way to check in with colleagues, *“it’s always much less visceral compared to having these conversations in person.”*

Working at a distance meant that naturally flowing conversations could prove to be a challenge. As P.13 explains this is *“because so much more ‘effort’ goes into communicating and it feels more formal than having face-to-face conversations.”*

When at a distance, being ‘invisible’ meant not being able to see if colleagues were okay, and also made it difficult to interpret their communication online. For example:

“…there was one of my office colleague who responded very rude to one of my query, I did wonder, was it working in loneliness that made my colleague to behave in the strange manner.” (P.6).

Some participants were cautious in terms of the ‘rules’ of communication: when to contact colleagues, worried they would be bothering them, ensuring everyone has a chance to speak in group calls and ensuring work does not ‘hijack’ social time. For example:

“The Microsoft Teams meeting was set up with the intention of social interaction. However, it was mostly work-related. Just before the end we talked about some private things.” (P.26).

#### Negatives Outweighed by WFH

The multiple dependency on technology during the pandemic – for work, for social interaction with colleagues and friends/family, for entertainment, etc. - resulted in significant time being spent on technology and was the cause of communication overload. Some colleagues were happy with communicating online and sometimes missed face-to-face interaction. Others missed the social aspect, stating *“it was boring working at home… It feel a bit less productive working at home…” (P.9).* Others, who did miss face-to-face interactions, weighed up the benefits in favour of WFH due to no longer having to travel to work. For example:

“Sleeping time adjusted because I no longer have to wake up too early to commute. It outweighs the challenges for me.” (P.19).

“I do not miss the ‘norm’. But I started to think about the things I did at work that I enjoyed and miss: 1.The staff member you encounter at the kitchen or the hallway and have a chat with which I don’t do remotely unless there is a reason for, 2.Going to the gym at lunch time and have a quick chat at the changing room, 3.The person you consistently encounter on the way to the office…you would exchange greetings or a joke or a nice gesture that makes you feel nice… On the other hand, I have much longer time with my family.” (P.2).

In terms of feeling socially involved or isolated, how participants felt varied from day to day, which highlights the importance of developing a communication strategy for yourself:

“One interesting thing for me is that getting the level of communication right while working remotely is quite difficult. Some days I’ve felt more isolated due to not very much communication, others I’ve felt really positive after a few check-ins and a Skype meeting, and sometimes I’ve felt overwhelmed or exhausted with too much communication and felt that it’s interrupted my workflow.”(P.13).

“MY OVERALL FEELING ABOUT BEING MORE ISOLATED MAY BE A LITTLE ODD. I MISS SEEING AND HUGGING PEOPLE BUT I ACTUALLY LIKE MY WORKING HOURS MORE… PARTIALLY BECAUSE IT IS MORE CONVENIENT FOR ME WITH MY LARGE FAMILY …I HAVE FIGURED OUT WAYS TO FEEL CONNECTED WITH PEOPLE ON-LINE. I MAKE SURE TO ENGAGE EACH PERSON ON THE CALL IN ONE WAY OR THE OTHER: ASKING THEM A QUESTIONS, FIXING THEIR FORM, DEDICATING A SONG TO THEM, MAKING FUN OF THEM (HEHEHEE!)… SO…. I LIKE THE NEW NORMAL.” (P.16).

### Beyond WFH – Returning to the Workplace

Three of the participants had the opportunity to visit their office during the time they were keeping their diaries. They commented:

“Today I went to office on need basis, though limited folks critical for project only could come to office, but felt good being in office premise and working at comfort of office desk. And it was productive also than regular days. Being in office without wasting time on office chit chat ☺ (as no one to chit chat on politics or social issues)” (P.6).

“Today I went to the office and I was the only one who is working from the office in my team. It was quite productive because there were only few people in the office so less socialising more work ☺ I believe it will be hard to return our “usual office life” because during the confinement, everybody got used to work alone and when there are people talking around you, it is really hard to focus on your work. Especially when half of the team works from home, almost everyone is on the phone via teams all the time so open offices got little bit noisier. If everyone was in the office, not everybody would be on the phone at the same time and when colleagues want to meet, they could use the meeting rooms. However, right now, there are not enough meeting rooms separately for everyone who is in the office for their online meetings.” (P.7).

Participant 19, who held a senior managerial post in his organisation, had been invited for a formal face-to-face meeting with other senior managers. He described that the meeting was “theatre-style seating, no one could see one another as everyone was facing in one direction” and that the person leading the meeting was not able to get their message across as no one could really see everyone else and because they were socially distanced.

Clearly, two respondents (P.6 and P.7 above) were pleased with the productivity benefits of returning to the office when there were fewer colleagues around and feeling good returning to a dedicated workspace. The experiences of Participants 7 and 19 highlighted the potential problems that may arise as more colleagues return to the office and the actual seating/practice changes that would be required in order to facilitate in-person collaborative activities.

The next section will provide a discussion of the key findings in relation to the literature.

## Discussion

It is clear from the participants’ experiences that organisations and employees have managed to adapt working practices to function remotely using collaborative tools such as Microsoft Teams and Zoom (Barnes, 2020; Nabity-Grover et al., 2020). The authors suggested that in response to, and in order to manage the negative implications of social distancing, individuals are spending more time on social media platforms. On the contrary, our findings show that only a minority of participants connected with colleagues on social media. With regards to how previously office-based workers now engage in social interaction with colleagues using technology, for which there is presently little empirical evidence, what is clear from the findings is that what social interaction means and how it takes place has changed: previously, corridor chats allowed for time to catch up on work and non-work information sharing. Now, social interaction becomes a part of a work conversation, fit around meetings for a few minutes. In line with Harris (2003), team meetings tended to be formal with limited scope for informal discussions. However, our findings suggest that the lack of social interaction is not to say that the participants did not want to interact with one another on a social level. Despite the fact that social interaction now often took place before/after meetings and for a lesser amount of time, participants were able to discuss various topics such as family, wellbeing and future holidays. Participants did not regard this as an issue, but every participant bar one did mention in their diary entries over a period of ten working days that they missed some form of in-person social interaction which entailed light conversation and some natural positive interaction.

It can be seen from the findings that the degree to which participants missed social interaction varied and depended on various factors. In line with Richter (2020), employees had experimented with virtual coffee meetings and after work social events via video calls. However, our findings highlight that very few homeworkers engaged in such activities: those who stated they missed in-person interaction with colleagues the most tended to be the ones who organised more social interactions online with colleagues such as online coffee chats and quizzes, and non-work-related discussions that would have occurred during natural breaks in the workplace, such as at lunch times. In addition, our study highlights that in some instances, where efforts were made by the organisation to involve employees in social activities, these had either faded in popularity or some participants were reluctant to participate. Reasons for this stated by participants included: (i) a high workload which meant they had no time to join the activities, (ii) scepticism about management wanting to socialise with employees when this did not happen offline, and (iii) too many hours already spent using technology for work purposes. For instance, half of the participants stated that they had high levels of work-related interaction with colleagues on a daily basis, often in back-to-back video calls. Thus, the desire to spend more time on a technology platform for the purpose of social interaction would not be very appealing, especially when participants had a heavy workload and worked in an atmosphere of uncertainty due to redundancies and lay-offs, and with negative impacts on their health.

Papagiannidis et al. (2020) stated that since video conferencing platforms are widely available, most users would not have faced major issues using these. However, our study suggests that it is not a question about usability, but that of the heavy reliance and usage of video conferencing platforms. The findings demonstrate that despite being seen as an attractive method of communicating, video calls were described as being ‘draining’ and, owing to the variety of platforms on which participants could be contacted via, this could also prove to be overwhelming. Davison (2020) suggests turning off the video during a video call can help individuals in meetings as it reduces the number of cues they need to process as well as noticing video-audio lags, even though some of the paralinguistic cues would disappear. However, our findings highlight that for the majority of participants, remaining connected via video and audio was mandatory in meetings which would often consume much of the working day. Thus, remaining receptive and perceptive to others’ demeanour and expressions explains the feeling of being ‘drained’, together with the fact that due to the long hours spent in online video conferences, a number of participants were less productive during the working day and subsequently had to work in the evenings in order to make up for the time lost in online meetings.

The findings of this study also suggest that despite being able to see and hear other video call participants, online interactions allowed for more miscommunication and misinterpretation of messages, the inability to convey feelings to colleagues and discussion of sensitive topics such as career progression, and the absence of emotional intelligence to judge how people are. In line with Kodama (2020), the findings of this study suggest that video communications/conferencing is a high-potential method of communication that is very useful for conveying image information. However, the findings further suggest that in video calls, especially in the context of large group meetings, the ability to gauge personal expressions is limited and the absence of emotional intelligence makes it difficult to ‘read’ people and situations as well as they could have been in-person. This, in turn, produces uncertainty regarding what to communicate and how to interpret the wellbeing of the potential recipient of a message.

There is no doubt that technology can facilitate communication. However, it is not just about the technology: there is so much more to it. Much like work practices that have adapted to WFH, communication practices in relation to social interaction can be considered in the same way. Organisations and individuals are trying different ways to connect. For instance, becoming part of a WhatsApp group created for the purpose of light-hearted entertainment can become overwhelming when you are trying to understand other group members’ personalities via short messages. Subsequently, the purpose of light-hearted banter is defeated. In line with Lal and Dwivedi (2009), what still appears to be key is being a part of a small network of close colleagues to be able to catch up with news, gossip and other information. Not being a part of a social circle in the workplace has been highlighted as a potential problem for new colleagues who have not been able to develop connections with other colleagues: this was something observed by participants who had new members join during/just before the lockdown. From the perspective of someone who has not been working in the organisation for long, this too was perceived to be a problem as there was no one to talk to at the end of a difficult day or when questions remain unanswered.

Interactions via technology are not like naturally flowing conversations in-person: this study emphasises that effort has to be put into communicating which means individuals would have to think carefully about what content to discuss. Specific social interaction had to be planned which is in contrast to previous impromptu interactions in the office, resulting in less informal conversations during the day and a more serious feel to the day which becomes more work focused. It was apparent from the findings that the ‘rules’ and norms of communication were unclear, such as ensuring everyone has a chance to speak in group calls and ensuring work talk does not creep into meetings specifically set up for non-work discussions. Also, the sender of the message thought carefully about the best mode of communication when contacting colleagues, worried they would be bothering them whereas in the office, people traffic was the norm. Furthermore, engaging online could lead to silences which can lead to feelings of awkwardness. This was made perhaps more uncomfortable for some considering the fact that it was mandatory for the majority of participants to use video and that half of the respondents said they experienced technology-enabled communication overload. Even if individuals in an online video meeting could read others’ expressions and tone of voice well, it is debatable whether this would actually matter. For example, if someone is clearly exhausted having sat through hours without a break, would anyone actually tell them they look terrible and need to take a break or would they have a virtual stiff upper lip? Such considerations all help develop our understanding of how homeworkers are beginning to adapt to remote working and communication. What is clear is that we cannot assume that in-person communication practices can be replicated using technology.

Since none of the participants had previously worked as full-time homeworkers prior to the pandemic, it was evident from their diary entries that they had both ‘good days’ and ‘bad days’ in terms of how they felt working from home. What was apparent in the diary entries is that participants were weighing up the benefits of working from home and the negative impact that WFH had on their social relationships with colleagues. Despite the drawbacks, for the majority of participants, the benefits of WFH far outweighed the negatives. However, although this may be acceptable now, in the long-term organisations have to think about the wider implications of reduced face-to-face interaction. For instance, the findings of this study show that in some cases, co-inhabiting others became the ‘new colleagues’ with whom participants spent break times with. These findings are in line with the study by Lal and Dwivedi (2009) which found that homeworkers would seek social interaction with family members to compensate for the lack of social interaction with colleagues. However, there are questions over for how long this will suffice.

Moreover, there are additional challenges for organisations while individuals continue to work at a distance from one another such as the challenge of maintaining the organisational culture (Barnes, 2020). Likewise, if homeworkers feel unsupported, this may result in a detrimental effect on trust and relationship with their employer, resulting in reduced collaboration for problem-solving, feeling out of the loop and increased staff turnover (Harris, 2003). Increased use of technology can also reduce feelings of belonging which is necessary for creating loyalty to both colleagues and the organisation (Mann & Holdsworth, 2003); thus, the longer the absence of face-to-face interaction, the more likely there will be an adverse effect on employees’ loyalty and employees’ feelings of insecurity. This is in addition to reduced interpersonal networking, communication and camaraderie, and less opportunity for sharing work-related information which affect employees’ ability to create identification with the company (Lal and Dwivedi, 2009). ‘Work’ has been, for some time, an activity that is carried out predominantly at a specific place within a specific time and involves one-to-one conversations, brainstorms, laughter and good cheer (Davison, 2020). However, as the findings of this study demonstrate, during the pandemic it has become a lot more serious, so much so that some participants viewed the return to the traditional office as something potentially problematic as social interaction was being viewed as a distraction from their ability to be more productive. Considering that WFH is expected to continue for some time into the future, it is all the more important to understand how employees are affected and what management can do to support them.

In summary, and in line with the research findings, discussion, and overall research objectives - (i) to explore the methods used to retain social interaction, and (ii) to identify any challenges/hindrances to social interaction when working from home – the following framework is proposed:

**Fig. 1:**
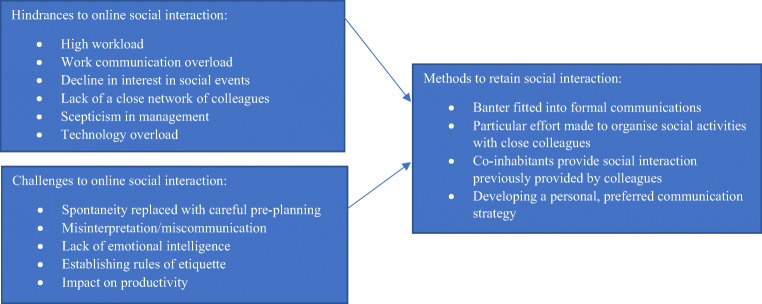
Social interaction when WFH

### Theoretical Contributions

Existing literature recognises that social interactions, which are typically conducted in the traditional workplace, are a fundamental feature of social life and the absence of such interactions can lead to feelings of social isolation and other negative repercussions. What is recognised in the literature is that there is a negative impact on social interaction and individuals WFH can often feel socially isolated. However, how homeworkers are able to maintain their social interactions with colleagues via technology when working remotely and the methods used to retain social interactions is under-researched. This study addresses these shortcomings in the literature and, given the prominence of WFH and the uncertainty of when individuals will return to their ‘normal’ work environments, this study has been valuable in providing insights into a variety of perceptions and feelings on how work has changed via an increased use of technology and the meaning, significance and scope for social interaction when WFH. The empirical evidence provided in this study also highlights the various reasons as to why individuals cannot/do not engage in social interactions via technology, the particular challenges related to online social interaction and how individuals maintain social interactions with colleagues: presently, there is limited understanding of these factors within the literature. Given that the consequences of the difficulties relating to the pandemic are expected to be longstanding - such as the wellbeing of individuals (Prime et al., 2020) – and that more job openings in the pandemic have allowed for WFH than before the pandemic (Koch et al., 2021), it is evermore important to develop an understanding of the practical issues faced by workers when WFH, thus making this study valuable.

### Implications for Practice

As well as contributing to the existing body of literature on homeworking and employees’ experiences and practices around social interaction with colleagues, this study has also highlighted the need for organisations to be aware of these differences and the highly contextualised nature of the perception of the opportunities and limitations of working from home. In this still dynamic situation of Covid-19, it is unclear what working life will look like in several months’ time. What is clear, however, is that organisations need to be aware of the different contexts in which they operate – what some would call organisational culture – and the different contexts in which their staff exist in order to ensure effective and supportive processes are in place. Furthermore, it is useful for organisations to be aware of other issues raised in the findings of this study: the reluctance to return to the ‘old normal’ workplace wherein social interactions are viewed as a distraction from their work as opposed to providing opportunities for camaraderie, motivation and teamworking.

### Limitations and Further Research

Considering the scale and speed at which WFH has been employed globally owing to the pandemic, at the time of this study the novelty/newness of this phenomena meant that changes in working practices and communications for homeworkers was all very new: the data was collected in May – June 2020 which was approximately 2–3 months after the shift to full-time WFH was made. Thus, it was the period when the dust was beginning to settle in the sense that individuals were adjusting to the new situation they faced themselves in and figuring out how to manage work from home. The open and exploratory approach therefore suited this study in developing initial understanding of how homeworkers were adjusting to their situation. Now, given that it is fast approaching one year since the shift to WFH occurred, and as homeworkers and their organisations are likely to have developed routines/norms/practices with regards to work, it would be advisable for future research to use theories such as the Normalisation Process Theory proposed within the Sociology discipline which can help to understand how individuals/actors engage with activities and the processes by which practices become routinely embedded in existing, socially patterned knowledge and practices (May & Finch, 2009). Thus, a more detailed understanding of the actions of homeworkers, the implications of WFH and the changing nature of work can be garnered. In addition, Fig. 1 is a first-attempt at understanding the factors in play with regards to a very actor-driven activity – social interaction – so it would be interesting to examine whether such factors are still present or whether other factors arise, given that WFH has now been mandatory for the best part of one year.

In terms of limitations of the study, it can be argued that using a snowball sample inhibited the range of responses gained in this study. Hence, future studies should consider alternative sampling techniques where a wider response can be gained so to include participants from a range of age groups: 22 out of 29 respondents in this study were aged between 25 and 44 years of age. Furthermore, the results of this study can be explored further by a more large-scale piece of research, taking into account the findings and testing them via a quantitative survey. It is also worthwhile to do a cross-cultural comparison – this would be one important way to further explore contextual differences within the data on working from home and social interaction given that a large percentage of the workforce across the world have been working from home for a significant amount of time now.

## Conclusions

This study has highlighted the myriad challenges and benefits of an increase in WFH exacerbated by Covid-19. Alongside changes in working practices and processes, there are clearly changes in communications practices. Social interaction, an integral part of many individuals’ day-to-day working lives, continues during WFH but at a much lesser extent and in a more organised way. Impromptu social interactions are replaced with pre-organised meetings where – in place of free-flowing conversation – the content is carefully considered: what social interaction means and how it takes place has clearly changed. Light-hearted banter and social interactions now largely take place before/after meetings. As this study highlights, despite attempts by organisations to host social events online, these had declined in popularity. Owing to the increased intensity that appeared to be characteristic of most participants’ daily working lives since the shift to full-time working from home, it was clear that while there were commonalities in experiences, there were also differences depending on context. A commonly shared belief was that so many hours per day were being spent on video calls that additional desire to also interact socially via the same medium was limited. Particularly, video calls were widely used and it was not just about being present, but involved processing various cues of various respondents which could be deemed as draining. Other factors such as uncertainties in terms of redundancies, concerns about health owing to long working hours and the pandemic, issues relating to miscommunication and misinterpretation in online interactions, the absence of emotional intelligence, concerns over career progression, communication overload via informal social media groups that were created with good intentions, the importance of having a circle of close colleagues, the lack of clarity on the rules and norms of communication etc. are also pertinent factors when considering the experiences of homeworkers and how they were able to adjust to this new way of working. Given that a significant number of organisations and employees have had to make the sudden change to working from home, and given the complex contexts in which organisations and employees work/exist, this study highlights that there are numerous issues that require further exploration both from the theoretical and practical perspectives in order to understand and ensure that WFH is a viable, manageable and fair mode of working.
